# Comparison of Leading Biosensor Technologies to Detect Changes in Human Endothelial Barrier Properties in Response to Pro-Inflammatory TNFα and IL1β in Real-Time †

**DOI:** 10.3390/bios11050159

**Published:** 2021-05-18

**Authors:** James J. W. Hucklesby, Akshata Anchan, Simon J. O’Carroll, Charles P. Unsworth, E. Scott Graham, Catherine E. Angel

**Affiliations:** 1 School of Biological Sciences, Faculty of Science, University of Auckland, Auckland 1010, New Zealand; 2 Department of Molecular Medicine and Pathology, Faculty of Medical and Health Sciences, University of Auckland, Auckland 1010, New Zealand; a.anchan@auckland.ac.nz (A.A.); s.graham@auckland.ac.nz (E.S.G.); 3 Centre for Brain Research, University of Auckland, Auckland 1010, New Zealand; s.ocarroll@auckland.ac.nz; 4 Department of Anatomy and Medical Imaging, Faculty of Medical and Health Sciences, University of Auckland, Auckland 1010, New Zealand; 5 Department of Engineering Science, Faculty of Engineering, University of Auckland, Auckland 1010, New Zealand; c.unsworth@auckland.ac.nz

**Keywords:** ECIS, xCELLigence, cellZscope, hCMVEC, endothelial cell, impedance sensing

## Abstract

Electric Cell-Substrate Impedance Sensing (ECIS), xCELLigence and cellZscope are commercially available instruments that measure the impedance of cellular monolayers. Despite widespread use of these systems individually, direct comparisons between these platforms have not been published. To compare these instruments, the responses of human brain endothelial monolayers to TNFα and IL1β were measured on all three platforms simultaneously. All instruments detected transient changes in impedance in response to the cytokines, although the response magnitude varied, with ECIS being the most sensitive. ECIS and cellZscope were also able to attribute responses to particular endothelial barrier components by modelling the multifrequency impedance data acquired by these instruments; in contrast the limited frequency xCELLigence data cannot be modelled. Consistent with its superior impedance sensing, ECIS exhibited a greater capacity than cellZscope to distinguish between subtle changes in modelled endothelial monolayer properties. The reduced resolving ability of the cellZscope platform may be due to its electrode configuration, which is necessary to allow access to the basolateral compartment, an important advantage of this instrument. Collectively, this work demonstrates that instruments must be carefully selected to ensure they are appropriate for the experimental questions being asked when assessing endothelial barrier properties.

## 1. Introduction

Impedance sensing is a label-free, real-time technique used to monitor cellular function. First pioneered by Giaever and Keese, impedance sensing exposes live cells to very small electrical currents across a range of frequencies [1,2]. By measuring the impedance that the cells provide to this current, we can accurately measure the responses of the cells in real-time. As no labelling is required, measurements are non-invasive and can be carried out over extended periods to give high-resolution information in real-time [3,4]. Furthermore, this information is inherently quantitative and thus can be readily analysed statistically [5,6]. Mathematical models can also be applied to this data to allow the exploration of various cellular parameters that cannot be measured directly [7]. These advantages have triggered the broad adoption of impedance sensing in a wide variety of applications, with a range of custom instruments having been developed [8,9,10]. However, the adoption of these systems has been limited, as the construction of customised specialist instrumentation is technically challenging. In contrast, commercially available instruments provide a turnkey solution to accessing impedance sensing. There are, however, only a few commercially available instruments including the Electrical Cell-Substrate Impedance Sensing (ECIS), xCELLigence and cellZscope platforms [11,12,13]. Despite the widespread use of these platforms individually to assess endothelial barriers [3,14,15,16,17,18] a systematic comparison of each platform’s capacity to resolve changes in endothelial barrier properties has not been conducted. Therefore, in this paper, the ability of these instruments to detect changes in endothelial barrier properties in response to TNFα and IL1β were compared.

**Figure 1:**
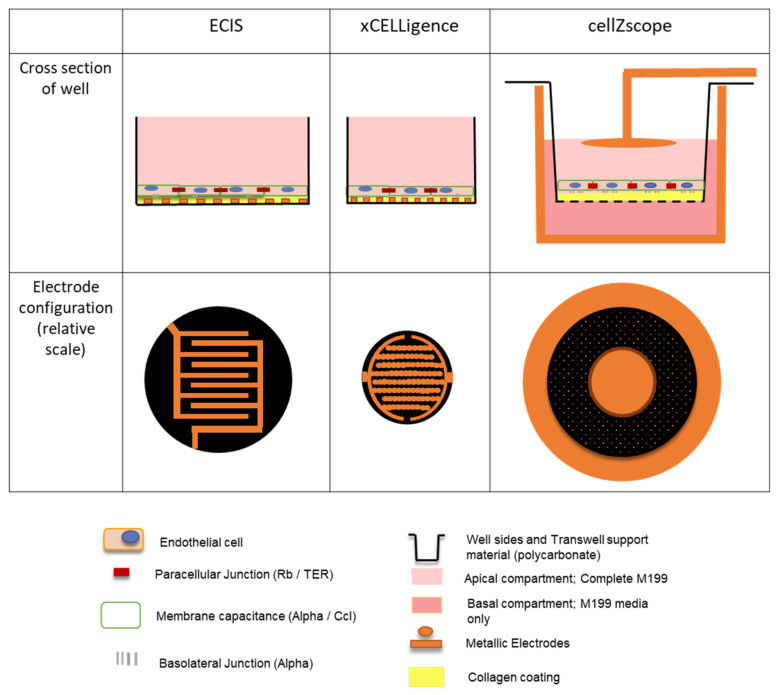
The electrode arrays used most widely in the ECIS (96W20idf plate), xCELLigence (E-plate) and cellZscope instruments differ in their electrode configuration. Both the ECIS and xCELLigence electrodes have a similar interdigitating electrode configuration, which covers a high proportion of the bottom of the well. Hence, their electrodes are directly coated with collagen and in intimate contact with the endothelial cells. In contrast, one of the cellZscope electrodes lines the lower compartment of the Transwell, whilst the second is suspended above the cell monolayer. Therefore, these electrodes are not in direct contact with the endothelial cells.

Giaever and Keese’s original Electric Cell-substrate Impedance Sensing (ECIS) invention has since been commercialized by Applied BioPhysics [11]. One such instrument is the ECIS ZΘ, which can be configured to measure cellular impedance in 96-well plates with gold electrodes fabricated directly onto the base of each well that has a growth area of 0.32 cm^2^ (Figure 1 and Supplementary Table S1). Impedance and phase measurements at frequencies ranging from 10 Hz to 10^5^ Hz are collected by the instrument (Supplementary Table S1). Subsequently, these can be modelled computationally to indicate biologically relevant cellular parameters. Three key values are generated: Rb, Cm and Alpha (Supplementary Table S1). Rb represents the cell–cell contacts, such as those formed by junctional molecules; Cm represents the membrane resistance of the cells; whilst Alpha represents the basolateral adhesion, which is influenced by both the distance between the cells and the underlying substrate and the presence of any junctional molecules bridging this interface [7]. Together, these values allow for the in-depth analysis of biological responses [19].

More recently, ACEA Biosciences (now part of Agilent) released the xCELLigence instrument [12]. Much like ECIS, this instrument uses gold electrodes fabricated directly onto the base of wells in a 96-well plate; each well has a growth area of 0.196 cm^2^ (Figure 1 and Supplementary Table S1). However, this instrument only collects impedance measurements at three frequencies, 10, 25 and 50 kHz (Supplementary Table S1). Although modelling cellular parameters is theoretically possible using three frequency measurements, the limited range of readings makes any results unreliable.

Finally, cellZscope is the most recent addition to the market, and is able to measure impedance across a Transwell filter with a cell growth area of 0.33 cm^2^ (Supplementary Table S1) [13]. The Transwell is seated in a stainless steel pot that acts as an electrical conductor. A second electrode suspended over the cells makes contact with the media in the apical chamber, completing the circuit and allowing impedance to be measured (Figure 1 and Supplementary Table S1). Like ECIS, phase and impedance data are collected at a range of frequencies from 1 Hz to 100 kHz and hence, can also be modelled (Supplementary Table S1). This results in the calculation of transepithelial-endothelial electrical resistance (TER) as a measurement of the cell–cell junctional interactions, and C_CL_ as a measure of cell layer capacitance (Supplementary Table S1) [13]. An equivalent of the Alpha value generated by the ECIS instrument is not included in this model, as the porous nature of the Transwells means that this parameter is not physically present and therefore not appropriate to infer.

Despite numerous studies using these instruments, direct comparisons between them have not been conducted. This is a critical lack of knowledge, as the inferences from the data collected from all three instruments are regularly used together to interrogate cellular responses [17,20,21,22,23]. Therefore, in this paper, we analyse the similarities and differences between these three commercially available instruments. The hCMVEC cell line was chosen due to its low overall resistance, which, although characteristic of brain microvascular endothelial cell lines [23], dictates the use of more sophisticated and more sensitive instrumentation [19].

The inflammatory cytokines TNFα and IL1β were selected for these experiments due to their well-defined biphasic response in this cell line. The response of hCMVECs to IL1β and TNFα has been explored at a molecular level and has been well-characterized using impedance instruments [17]. These responses are ideal for this study, as the cytokines first cause a decrease in resistance, followed by a substantial increase for an extended period. Therefore, both decreases and increases in resistance can be examined with the same stimulus. The transient initial decrease in resistance also showcases the high time resolution of impedance sensing, by highlighting a response that could easily go undetected between the time points of a traditional end-point assay [24]. For this study, TNFα and IL1 β concentrations were selected to provide a robust biphasic response with which to test the impedance instruments [17].

We evaluated two key parameters of the data produced: the difference in magnitude at key points in time, and the profile of the temporal measurements resulting in different curve shapes. A difference in magnitude is informative, straightforward to interpret and correlates with traditional single-time point assays [19,20]. The second characteristic, the profile of the temporal measurements or shape of the curve, is also useful. Even if two responses have the same magnitude at a key time point, they may reach that point in a very different way. This characteristic was analysed using cross-correlation with no lag, which generates a single value between 1 and −1 for each pair of curves. A value of 1 represents identical curves, 0 shows no correlation between the curves and −1 represents curves with a mirror image opposing profile or inverse correlation [25]. By assessing the magnitude and temporal profile of the response in concert, we are able to rigorously compare the measurements from all three instruments.

In this study, we ran the same experiment simultaneously on the three impedance-sensing instruments. We show that, although the instruments’ temporal impedance measurements have similar profiles, they differ in magnitude, demonstrating significant differences in sensitivity. Furthermore, the modelled data reinforces the differences in sensitivity between the instruments and reveals changes in endothelial barrier properties that were not evident from the overall impedance measurements. Together, this demonstrates the importance of selecting the most appropriate instrument for a particular study.

## 2. Materials and Methods

### 2.1. Culture of Human Brain Endothelial Cells

Human cerebral microvascular endothelial cells (hCMVECs) were purchased from Applied Biological Materials Inc (cat# T0259). hCMVECs were cultured in 75 cm^2^ (T75) Nunc flasks (cat# 156499) with M199 medium containing 10% FBS, 1 μg/mL hydrocortisone, 3 ng/mL hFGF, 1 ng/mL hEGF, 10 μg/mL heparin, 2 mM GlutaMAX and 80 μM dibutyryl-cAMP (later referred to as complete M199 medium) at 37 °C, with 5% CO_2_ and 100% humidity. For both hCMVEC maintenance and experiments, culture vessels were coated with 1 μg/cm^2^ collagen I dissolved in 0.02 M acetic acid for 1 h at room temperature, before being washed 3 times with sterile MilliQ water and seeding the hCMVECs. To passage the hCMVECs, T75 flasks were washed twice with 4 mL pre-warmed PBS before being incubated with 4 mL pre-warmed TrypLE for 5 min at 37 °C. The TrypLE activity was then neutralized with 4 mL complete M199 and the cells were centrifuged at 100× *g* for 5 min, counted, and seeded for experiments. All experiments used hCMVECs between passages 11 to 16. All impedance instruments and experimental hCMVEC cultures were kept in dedicated incubators at 37 °C, with 5% CO_2_ and 100% humidity.

### 2.2. Impedance Sensing Experiments

ECIS: 96W20idf plates were treated with 10 mM cysteine for 15 min to clean the electrode and standardize the electrode impedance (as per manufacturers’ instructions). The wells were then coated with collagen as described above. The hCMVECs were seeded in 200 µL complete M199 medium. The ECIS machine was run continuously in multi-frequency mode using the default frequency spectra (Supplementary Table S1).

xCELLigence: E-plates (96 wells) were coated with collagen as described above. Complete M199 was added to each well and calibration was conducted. Cells were seeded in 122 µL Complete M199. Impedance was measured at 10, 25 and 50 kHz (Supplementary Table S1).

CellZscope: before the experiment, cellZscope components were cleaned with MilliQ water, 70% ethanol, and then MilliQ water again. The pots and dipping electrodes were autoclaved, whilst the remainder of the Cell Module was sterilised with 70% ethanol. Before coating, the Cell Module was assembled under sterile conditions, and each of the stainless steel pots was flooded with 900 µL basal M199 media. The assembled module was then placed in the cell culture incubator to equilibrate for at least one hour. Transwells (Corning; 6.5 mm insert, 0.4 µm pore size) were coated from the apical side, as previously described in Section 2.1. The hCMVECs were then seeded into the apical chamber in 200 µL complete M199 medium. Transwells were then transferred into the Cell Module, taking care not to trap any bubbles underneath the membrane. The Cell Module was then placed in the instrument, and the spectra were acquired at the highest resolution between 1 and 100 kHz (Supplementary Table S1). Measurements were made every 15 min, the fastest rate possible at these frequency settings.

### 2.3. Treatment with Inflammatory Cytokines

After seeding, the cells were cultured for 48 h to allow the barrier to fully develop and impedance to stabilise. On the day of treatment a 5× stock of TNFα and IL1β in complete M199 was prepared; once added to the corresponding culture wells this provided a final concentration of 500 pg/mL of TNFα or 500 pg/mL IL1β. For the control treatment, the 5× stock consisted of complete M199 with an equivalent amount of MilliQ water (henceforth labelled as the control). Each instrument was then paused, and the 5× stock was gently introduced to the middle of the well or apical chamber. The cultures were then returned to the respective instrument and the measurements resumed. Cell monitoring continued on all instruments for a further 27 h.

### 2.4. Data Analysis

Modelling was conducted against a cell-free well in the same experiment using software provided by the vendor for each instrument; ECIS Software (V 1.1.252, Applied Biophysics), RTCA Software (V 2.0.0.2301, AECA Biosciences Inc.) and cellZscope (V 4.3.4, nanoAnalytics) for ECIS, xCELLigence and the cellZscope respectively.

Graphs were generated using ggplot2 version 3.3.2 [26]. All experiments were conducted in triplicate and the mean ± standard error of the mean (SEM) from three independent experiments were plotted.

RStudio (version 1.1.414, RStudio, Inc., Boston, MA, USA) and vascr (developed by J. Hucklesby) [6] were used to generate the cross-correlation values. vascr uses the ccf function in the stats package to run the underlying cross-correlation analysis. No lag value was applied. Temporal response profiles for each experiment were generated by averaging measurements from three technical replicates. Cross-correlation results show the mean ± SEM of the values derived from the two temporal response profiles being compared, each of which includes data from three independent experiments.

## 3. Results and Discussion

**Figure 2:**
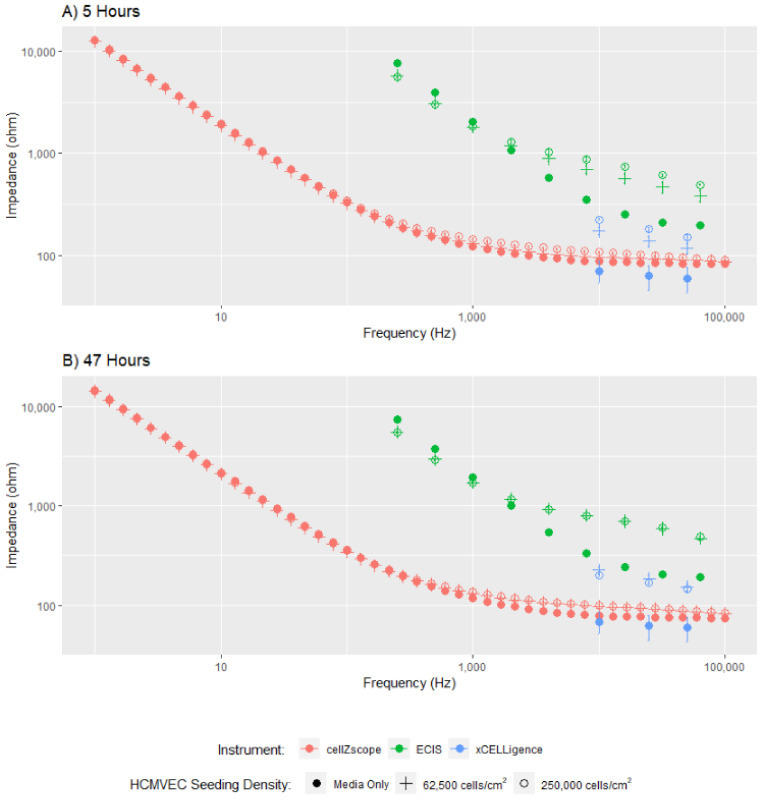
Impedance spectra of hCMVECs obtained using the ECIS, cellZscope and xCELLigence instruments. The impedance spectra of two different initial cell seeding densities were assessed at 5 and 47 h. Each point represents the mean ± SEM at each frequency where the impedance was measured. These data are derived from three independent experiments, each of which was conducted in triplicate.

To assess the comparability of the three instruments, we first collected the impedance spectra of a confluent hCMVEC monolayer at 5 and 47 h, after the seeding of either 250,000 cells/cm^2^, 62,500 cells/cm^2^ or media only (Figure 2). As all three platforms were seeded simultaneously using the same preparation of cells, we can directly compare the measurements collected.

The dataset in Figure 2 clearly demonstrates the quantity of data collected by each instrument and the relative concordance of the data obtained from these three instruments. The cellZscope captured 34 data points, compared to 9 from the ECIS instrument, and only 3 from the xCELLigence platform. For modelling to be accurately conducted, we require data showing the impedance response of cells over a large frequency range [2]; hence the small number of data points spanning a narrow frequency range that were acquired using xCELLigence cannot be accurately used to model different endothelial barrier properties. Therefore, only the overall impedance change obtained using the xCELLigence can be assessed, a value that incorporates several undistinguishable cellular parameters, limiting the interpretation of these data. The capacity of the ECIS and cellZscope instruments to model the data they acquire over a larger frequency range will be explored later in this study.

Despite variation in magnitudes between all three instruments, the cell impedance spectra generated by each instrument follow a similar trend, indicating that similar cellular characteristics are being measured. As expected, the impedance data for cell-seeded wells is higher than the media-only controls, indicating the cell monolayer has been detected. The difference between cell-seeded wells and the media-only control is subtle for the cellZscope data. This may be partly because the cells are not in direct contact with the electrodes, as they are in the ECIS and xCELLigence instruments (Figure 1 and Supplementary Table S1), meaning the current may be less concentrated when it passes through the cells resulting in a more subtle response. Collectively, the differences in magnitude observed between these data are likely due to variabilities in electrode area and configuration between the instruments (Figure 1 and Supplementary Table S1), which affects the absolute value of the impedance measured.

The dataset in Figure 2 also provides some insight into the temporal changes in impedance for the cells assessed using each of these three instruments. At the 5-h time point, there is a clear difference in impedance between the two cell seeding densities, however, at 47 h this difference is no longer apparent. This is because endothelial cells rapidly proliferate until they come into contact with neighbouring cells and form a monolayer, at which point proliferation slows and a mature monolayer forms [27]. By 47 h, the cells seeded at the lower density had sufficient time to proliferate and form a monolayer similar to the monolayer formed earlier by the cells seeded at a higher density.

**Figure 3:**
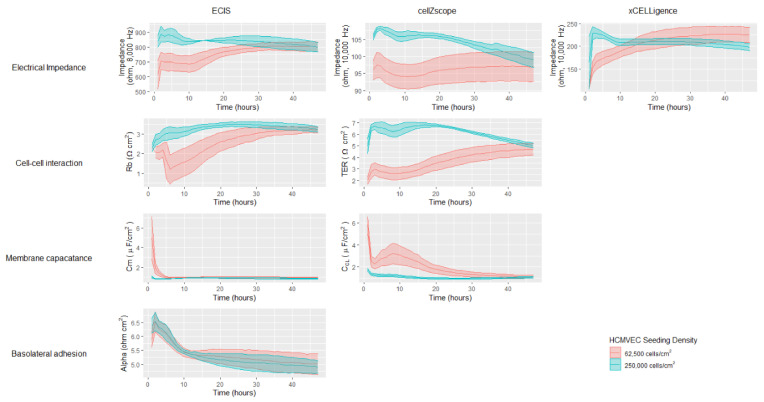
Temporal profile of impedance and modelled endothelial barrier properties of hCMVECs monitored over 48 h by ECIS, cellZscope and xCELLigence instruments. hCMVECs were initially seeded at either 62,500 cells/cm^2^ or 250,000 cells/cm^2^ and incubated for 48 h until confluent. Ribbon plots show the mean ± SEM of three independent experiments, each of which was conducted in triplicate.

To further explore the effect of cell seeding density on hCMVEC proliferation and monolayer formation, the measured impedances and modelled barrier properties were assessed over 48 h (Figure 3). Data from all three instruments showed that cells seeded at the higher density exhibited a high level of impedance at the start, which declined slowly and to varying extents during the 48-h period. In contrast, cells seeded at the lower density started with a lower impedance value that slowly increased and plateaued by approximately 40 h. Interestingly, the data acquired using the ECIS and cellZscope showed that the impedance values of the two seeding densities overlapped by 48 h, consistent with the endothelial cell growth properties discussed earlier. However, the data generated using xCELLigence showed that the impedance of the cells seeded at the higher density dropped below those seeded at the lower density by 48 h.

Impedance measurements provide useful insight into overall cellular and monolayer properties, however modelling impedance data acquired over a large frequency range can provide valuable information regarding more distinct cellular and barrier properties. As mentioned earlier it is not appropriate to model xCELLigence data that is generated using a limited frequency range. It is possible however to model the multifrequency data generated using the ECIS and cellZscope instruments (Supplementary Table S1). ECIS and cellZscope can provide Rb and TER values respectively, that use the entire impedance spectra to infer the extent of the cell-cell interactions that have formed (Figure 3). Modelled data for the lower seeding density from both instruments showed an increase in cell-cell interactions during the 48-h period; interestingly the cell–cell interactions modelled from the cellZscope data continued to increase after the impedance had begun to plateau (Figure 3). This shows that a plateau in impedance does not necessarily mean that the cell-cell interactions are fully formed. Membrane capacitance values were also modelled using the ECIS and cellZscope data; overall the hCMVECs exhibited low-level membrane capacitance (Figure 3). The cell membrane capacitance appeared to stabilise within the first 10 h on the ECIS instrument, however, it took a full 40 h for the cell membrane capacitance to stabilise on the Transwells in the cellZscope. ECIS was the only instrument capable of generating data that could infer the basolateral adhesive properties of the cells. This is because the cells are grown on a solid substrate, directly beneath which lie the electrodes. Meaning alpha, which represents basolateral adhesion, can be calculated as illustrated in the equivalent circuit diagram in Supplementary Table S1. In contrast, in the cellZscope the electrodes are not in direct contact with the cells or the solid substrate they are grown on and hence the basolateral adhesion can’t be modelled (Figure 1 and Supplementary Table S1). The ECIS profiles of the basolateral adhesive properties of both cell-seeding densities were similar, both declining and stabilising at approximately 12 h (Figure 3). Collectively the data generated by all three instruments using the lower cell seeding density indicates that a stable confluent monolayer had formed by 48 h; this consistency between instruments reflects their similar trends in impedance spectra (Figure 2). These data demonstrate the utility of these three instruments to assess cell growth and monolayer barrier properties, however, it does not interrogate their ability to assess temporal changes in response to biological stimuli.

**Figure 4:**
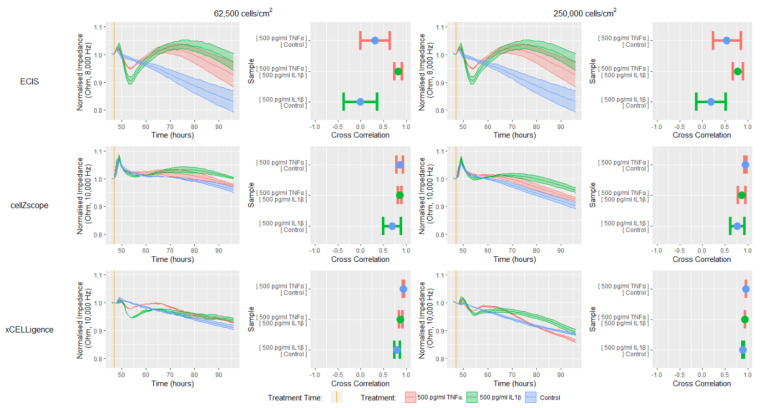
ECIS has a greater capacity to distinguish between different temporal impedance response profiles than cellZscope or xCELLigence. hCMVECs were seeded at either 62,500 cells/cm^2^ or 250,000 cells/cm^2^ and incubated for 48 h until confluent. The cells were then treated with TNFα or IL1β, and monitored using ECIS, cellZscope or xCELLigence for a further 48 h. The impedance data has been normalized at one hour before treatment and is presented as a change in impedance to allow direct comparisons between instruments to be made. Ribbon plots show the mean ± SEM of three independent experiments. Cross correlation results show the mean ± SEM of the values derived from the two temporal response profiles being compared, each of which includes data from three independent experiments. Cross-correlation is expressed as a value between 1 and −1, where 1 represents identical curves, 0 shows no correlation between the curves and −1 represents curves with a mirror image opposing profile or inverse correlation. N.B. Each treatment is indicated by the same colour in both the ribbon plots and cross correlation plots.

Next, each system’s capacity to detect temporal cellular changes in response to a biological stimulus was tested by treating, the confluent cell monolayers formed at 48 h with the proinflammatory cytokines TNFα and IL1β (Figure 4). The impedance data presented in Figure 4 has been normalized at one hour before treatment and is presented as a change in impedance, to allow direct comparisons between the instruments to be made. Finally, cross-correlation analysis was conducted between each treatment for all instruments; this analysis will test each instrument’s ability to discern between different temporal response profiles by comparing the curve shapes.

Each of the instruments were able to detect impedance differences between the control, TNFα and IL1β treatments, however, the ECIS instrument appeared to be the most sensitive showing the largest difference between treatments (Figure 4). The ECIS data showed that both IL1β and TNFα induced an initial rapid reduction in impedance, followed by a sustained increase that slowly declined after 70 h. These trends were apparent for both cell-seeding densities. Similar trends were also observed for the xCELLigence data, however, there was a reduced magnitude in both the responses detected and the differences between treatments, when compared with the ECIS data. The similar trends observed using these two instruments could be attributed to the similar interdigitating electrode configuration and the high proportion of electrode coverage on the bottom of the wells (Figure 1, Supplementary Table S1), meaning that both instruments can detect changes in endothelial monolayer impedance throughout a large proportion of the well. The ECIS instrument’s superior ability to resolve the temporal profiles of each of the proinflammatory treatments from the control was reflected by corresponding low cross-correlation values. In contrast, the higher cross-correlation values obtained for the analogous xCELLigence data reinforce this instrument’s reduced resolving capacity.

In contrast to ECIS and xCELLigence, the impedance data obtained using the cellZscope did not show a substantial difference between treatments during the initial 5 h, just the peak associated with adding a treatment (Figure 4). Thereafter, however, there was a slight increase in impedance following treatment with both TNFα and IL1β for both cell seeding densities, which slowly declined after approximately 65 h. Despite the subtle differences in cellZscope’s temporal profiles, the cross-correlation data indicated that IL1β appeared to influence the impedance of the endothelial monolayer to a greater extent than TNFα, a trend that was consistent for all three instruments. The reduced magnitude of the differences in the impedance temporal profiles observed with cellZscope, when compared with ECIS and xCELLigence may be a result of its distinct electrode configuration and the fact that the electrodes are not in direct contact with the cells or the substrate they are grown on (Figure 1, Supplementary Table S1). Collectively, these data demonstrate that the ECIS platform has a superior capacity to distinguish between the temporal impedance profile of a control endothelial monolayer and endothelial monolayers responding to TNFα or IL1β, when compared with the cellZscope and xCELLigence platforms.

**Figure 5:**
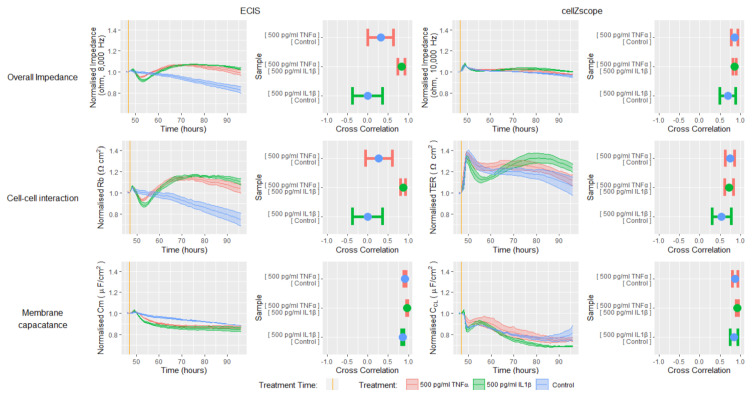
Modelling impedance data generated by ECIS or cellZscope reveals changes in endothelial barrier properties in response to TNFα or IL1β. hCMVECs were seeded at 62,500 cells/cm^2^ and incubated for 48 h until confluent. The cells were then treated with TNFα, IL1β or a vehicle, and the temporal profile of impedance was monitored using ECIS or cellZscope for a further 48 h. The impedance data were modelled to provide a temporal profile of cell-cell interactions (Rb) and membrane capacitance (Cm). All data has been normalized at one hour before treatment and is presented as a change in impedance, cell-cell interactions or membrane capacitance, to allow direct comparisons between instruments to be made. Ribbon plots show the mean ± SEM of three independent experiments. Cross-correlation results show the mean ± SEM of the values derived from the two temporal response profiles being compared, each of which includes data from three independent experiments. Cross-correlation is expressed as a value between 1 and −1, where 1 represents identical curves, 0 shows no correlation between the curves and −1 represents curves with a mirror image opposing profile or inverse correlation. N.B. Each treatment is indicated by the same colour in both the ribbon plots and cross-correlation plots.

To reveal the endothelial cellular and monolayer properties that are causing the temporal changes in impedance in response to TNFα or IL1β, we next modelled the data generated using the ECIS and cellZscope (Figure 5, equivalent circuits in Supplementary Table S1). These experiments were conducted using the lower cell seeding density as the data in Figure 4, and demonstrated that the magnitudes of the impedance responses were similar for each cell seeding density tested. The data in Figure 5 has been normalized and is presented as a change in either impedance, cell-cell interactions or membrane capacitance, to allow direct comparisons between instruments to be made.

The modelled Rb and TER values in Figure 5 represent the level of interaction that exists between neighbouring endothelial cells in a monolayer, for example, the junctional molecules and cell-cell contacts [7]. The TNFα and IL1β induced impedance profiles measured by ECIS and cellZscope were mirrored by the modelled Rb and TER profiles respectively, indicating that the cell-cell interactions between the hCMVECs contribute substantially to the overall impedance measurement. Both the ECIS and cellZscope data in Figure 5 indicate that IL1β stimulates an initial weakening followed by a sustained strengthening of cell-cell interactions, relative to the control. In contrast, the effect of TNFα on cell–cell interactions differ between instruments; ECIS shows that TNFα stimulates an initial weakening followed by a sustained strengthening of cell–cell interactions, relative to the control; whilst the cellZscope profile infers that TNFα does not weaken cell-cell interactions below that of the control, and the subsequent strengthening is slight, relative to the control.

The impedance data can also be modelled to indicate the capacitance of the endothelial membrane layer. The magnitude of the changes in capacitance in response to IL1β and TNFα for both instruments was small relative to the changes in cell-cell interaction, indicating that these cytokines influenced capacitance to a lesser extent than they did the cell-cell interactions. Interestingly, the capacitance profiles in response to TNFα and IL1β differs for each instrument; ECIS shows that both proinflammatory cytokines induce a reduction in capacitance relative to the control that stabilises at approximately 65 h, whereas the cellZscope indicates that neither TNFα or IL1β stimulate a change in capacitance relative to the control until approximately 65 h when IL1β induces a decline in capacitance.

Collectively, the magnitude of the differences in measured impedance and modelled data, between treated and untreated endothelial monolayers was greater for ECIS than it was for the cellZscope data. This increased sensitivity meant that the ECIS system was able to definitively distinguish between both proinflammatory treatments and the control temporal profile, which was reinforced by the low cross-correlation values generated from these comparisons. These observations confirmed earlier findings showing that both IL1β and TNFα can influence cell-cell interactions that contribute to endothelial monolayer impedance [17]. The reduced sensitivity of the cellZscope meant it was only able to distinguish between the IL1β and control profiles; therefore it appears that this platform may not be able to definitively resolve subtle changes in endothelial monolayer properties, such as the lesser TNFα response in this study. As mentioned previously, the reduced sensitivity of the cellZscope may result from the electrodes being distant from the monolayer culture and measuring the impedance of the culture as a whole (Supplementary Table S1). In contrast, the ECIS electrodes span a large proportion of the culture surface (i.e., 3.985mm^2^, Supplementary Table S1) and are therefore in direct contact with a high proportion of the cell monolayer and its underlying substrate. Collectively this widespread electrode coverage and direct culture contact likely contributes to the enhanced sensitivity of the ECIS platform.

Despite cellZscope’s reduced sensitivity, it is important to note that this platform provides access to the basolateral compartment, thereby enabling studies that either need to apply treatments from beneath the monolayer, want to generate and study stratified cultures or wish to assess the transport of cells or molecules across the monolayer. The cellZscope’s 24 well capacity also provides considerable scope for assessing multiple treatments in these types of studies. Although, if access to the basolateral compartment is not required, either the xCELLigence or ECIS platforms 96 well arrays may be more attractive for large-scale experiments.

## 4. Conclusions

The data presented in this study highlights that the instrument used to assess changes in endothelial cell monolayer properties should be carefully selected, to ensure it is appropriate for the experimental questions being addressed. Although both the ECIS and xCELLigence platforms can facilitate large-scale screening on 96 well plates with similar electrode configurations, the ECIS platform is more sensitive than xCELLigence when detecting impedance changes in response to a stimulus. Furthermore, ECIS can acquire data at multiple frequencies, which can be modelled to identify which of the endothelial barrier components contributing to impedance are being affected, something xCELLigence is unable to do because of its limited frequency acquisition range. The cellZscope instrument also acquires impedance data at multiple frequencies which can be modelled to identify changes in particular endothelial barrier components, however, the reduced sensitivity of this platform relative to ECIS means that subtle changes in endothelial monolayer properties may not be resolved to the same extent as they can be by ECIS technology. The reduced sensitivity of the cellZscope platform could be due to its distinct electrode configuration that allows access to the basolateral compartment, which is essential for certain types of experimental approaches. Ultimately, the choice of platform hinges on: (1) whether access to the basolateral compartment is required, (2) if the researcher wishes to identify which endothelial monolayer properties are being influenced and (3) whether a high degree of sensitivity is required to detect subtle changes.

## Data Availability

Not applicable.

## Supplementary Materials

The following is available online at https://www.mdpi.com/article/10.3390/bios11050159/s1, Supplementary Table S1: ECIS, xCELLigence and cellZscope instrument parameters.
