# Supplementary material for: Comparison of Leading Biosensor Technologies to Detect Changes in Human Endothelial Barrier Properties in Response to Pro-Inflammatory TNFα and IL1β in Real-Time
Source: Biosensors (Basel). 2021 May 18;11(5):159. doi: 10.3390/bios11050159 (PMC8157866; doi:10.3390/bios11050159)
Supplement: Supplementary file 1 [file biosensors-11-00159-s001.zip › biosensors-1212535-supplementary.pdf]

Supplementary Table 1. ECIS, xCELLigence and cellZscope instrument parameters

	**ECIS**	**xCELLigence**	**cellZscope**
**Frequency range**	Range: 10 Hz to 10^5 Hz<br><br>Exact values are user specifiable. The values used in this study were the default frequencies of 62.5, 125, 250, 500, 1 000, 2 000, 4 000, 8 000, 16 000, 32 000 and 64 000 Hz	Data can only be collected at three frequencies:<br>10 000, 25 000 and 50 000 Hz	Range: 1Hz to 100 kHz<br><br>Exact values are selected from either “coarse” or “fine”. Fine resolution was used in this study, corresponding to frequencies of 1.29, 1.67, 2.15, 2.78, 3.59, 4.64, 5.99, 7.74, 10.00, 12.92, 16.68, 21.54, 27.83, 35.94, 46.42, 59.95, 77.43, 100.00, 129.15, 166.81, 215.44, 278.26, 359.38, 464.16, 599.48, 774.26, 1 000.00, 1 291.55, 1 668.10, 2 154.43, 2 782.56, 3 593.81, 4 641.59, 5 994.84, 7 742.64, 10 000.00, 12 915.50, 16 681.01, 21 544.35, 27 825.59, 35 938.14, 46 415.89, 59 948.43, 77 426.37 and 100 000.00 Hz
**Modelled values**			
Cell-cell interaction	Rb	-	TER
Membrane capacitance	Cm	-	C_{CL}
Basolateral adhesion	Alpha	-	-

<table>
<tr><td>Equivalent circuits</td><td>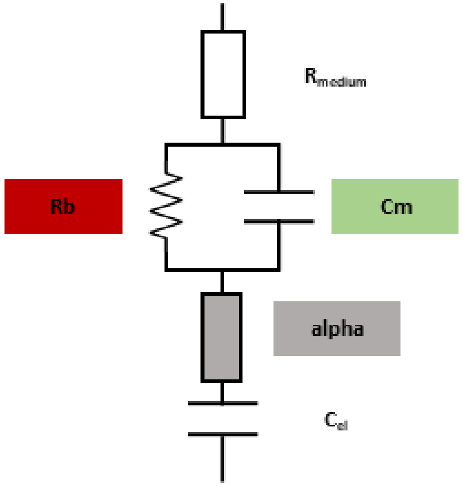
</td><td>Not determined, as modeling is not appropriate for this system</td><td>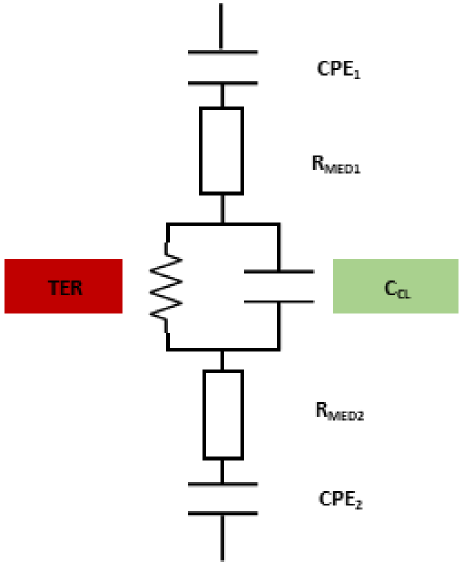
</td></tr>
<tr><td>Cell culture vehicle</td><td>ECIS 96 well plates (96W20idf plate)</td><td>xCELLigence 96 well plates (E-plate)</td><td>Transwells (available from various manufacturers) that fit in re-usable stainless steel pots</td></tr>
<tr><td>Electrode material</td><td>Gold</td><td>Gold</td><td>Stainless Steel</td></tr>
<tr><td>Well growth area</td><td>0.32 cm^2</td><td>0.196 cm^2</td><td>0.33 cm^2</td></tr>
<tr><td>Electrode area</td><td>0.3985 cm^2</td><td>~80% of cell growth area (~0.157 cm^2)</td><td>Not provided by manufacturer</td></tr>
</table>

**Photo showing top view of electrode configuration in each well (lid removed).**<br><br>Scale: ruler shows 1mm increments	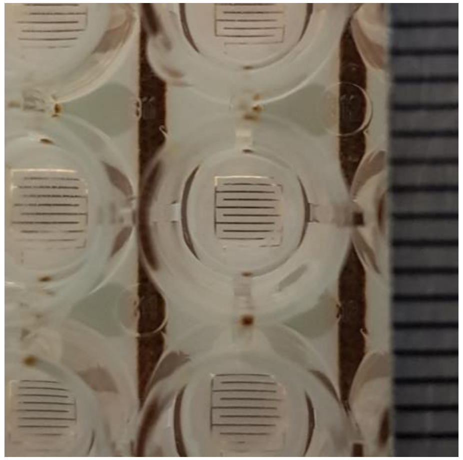	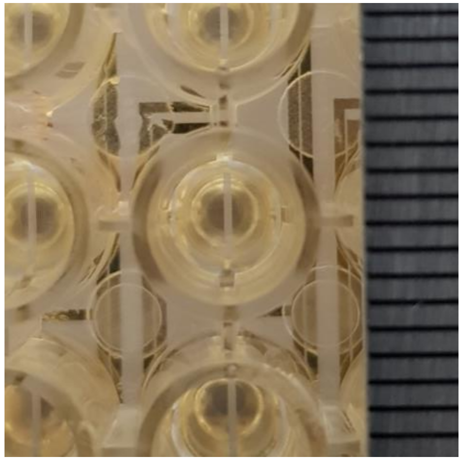	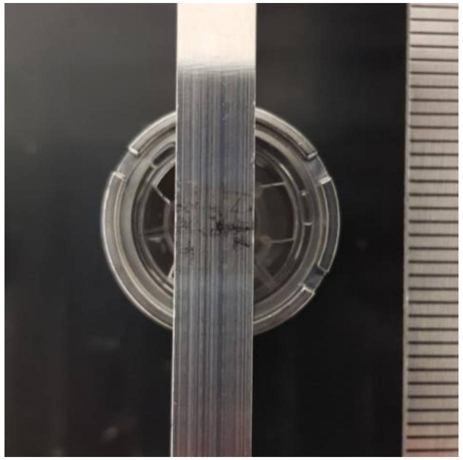
**Photo showing top view of entire plate or cell module used in each system.**<br><br>Scale: ruler shows 1mm increments	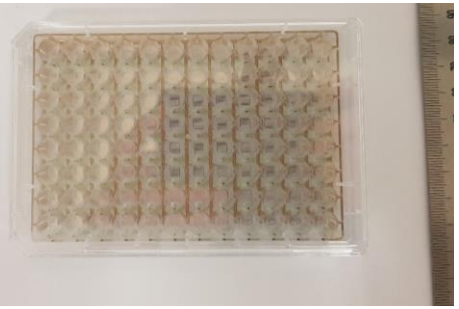	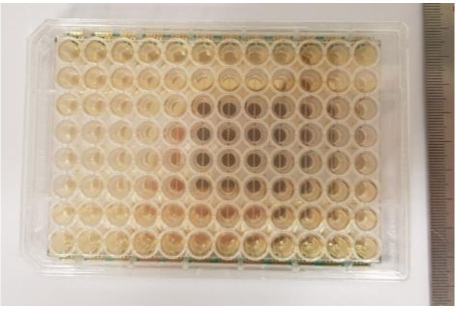	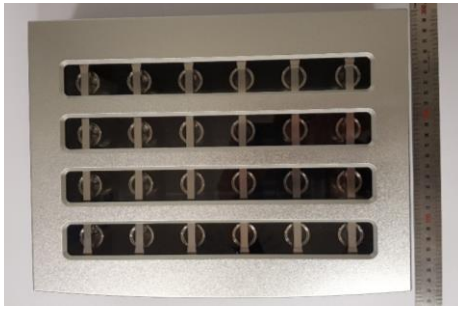

N.B. Some of the information provided in this table can also be sourced from the technical manuals for each instrument from their respective manufacturers.
